# Decreased T helper 17 cells in tuberculosis is associated with increased percentages of programmed death ligand 1, T helper 2 and regulatory T cells

**DOI:** 10.1186/s12931-017-0580-3

**Published:** 2017-06-26

**Authors:** Chin-Chung Shu, Ming-Fang Wu, Jann-Yuan Wang, Hsin-Chih Lai, Li-Na Lee, Bor-Luen Chiang, Chong-Jen Yu

**Affiliations:** 1 Graduate Institute of Clinical Medicine, College of Medicine, National Taiwan University, Taipei, Taiwan; 2 Department of Traumatology, National Taiwan University Hospital, Taipei, Taiwan; 3 Department of Internal Medicine, National Taiwan University Hospital, Taipei, Taiwan; 4 Graduate Institute of Toxicology, College of Medicine, National Taiwan University, Taipei, Taiwan; 5 Department of Medical Biotechnology and Laboratory Science, Chang Gung University, Tao-Yuan, Taiwan; 6 Department of Laboratory Medicine, National Taiwan University Hospital, Taipei, Taiwan; 7 Department of Pediatrics, National Taiwan University Hospital, Taipei, Taiwan; 8 Department of Medical Research, National Taiwan University Hospital, #7, Chung-Shan South Road, Taipei, 100 Taiwan

**Keywords:** Programmed cell death 1, Cytotoxic T-lymphocyte-associated protein 4, Tuberculosis, Th17 cells, Regulatory T cells, Th2 cells

## Abstract

**Background:**

Tuberculosis (TB) is one of the most common infectious diseases worldwide. During active tuberculosis, T helper (Th) 17 cells are decreased, however the association with inhibitory immune regulation is unclear.

**Methods:**

We enrolled 27 patients with TB and 20 age- and sex-matched controls and studies their lymphocyte status. Peripheral blood lymphocytes were isolated and programmed death-1 (PD-1) and programmed death ligand 1 (PD-L1) were measured on Th17 cells by using flow cytometry after the cells were stimulated with phorbol 12-myristate 13-acetate and ionomycin for 6 h. In addition, Th2 and regulatory T cells were measured and analyzed.

**Results:**

The TB group had lower levels of Th17 cells but higher levels of Th2 and Treg cells than the controls. In Th17 cells, the percentage of PD-L1 was higher in the TB group than that in the controls. In Th2 and Treg cells, the percentage of cytotoxic T-lymphocyte associated protein 4 (CTLA-4) was lower in the TB group and PD-1 was higher in Treg cells in the TB group. In the patients with extra-pulmonary TB, levels of Th1, Th2 and T17 cells were lower than those with pulmonary TB. The percentage of PD-1 on Th1 lymphocytes positively correlated with radiographic score.

**Conclusions:**

Lower level of Th17 in TB patients may be associated with increased percentage of PD-L1 and increasing levels of Th2 and Treg cells which influenced by CTLA-4.

**Electronic supplementary material:**

The online version of this article (doi:10.1186/s12931-017-0580-3) contains supplementary material, which is available to authorized users.

## Background

Tuberculosis (TB) is the most important infectious disease worldwide. According to the World Health Organization, 300 million people are infected with *Mycobacterium tuberculosis,* and 30 million people with TB died from 2001 to 2010 [1, 2]. Timely treatment is one of the most important strategies to prevent further transmission of TB [3–5]. In addition to clinical judgment, understanding the immune process during active TB is important for clinical prediction of outcome and search of future target therapy.

In the pathogenesis of TB, adaptive immunity plays a pivotal role in primary TB and its reactivation. T helper (Th) 17 cells are an important type of lymphocyte that can establish protective immunity to TB in addition to Th1 cells, and they have been shown to have a significant pro-inflammatory effect in protecting against intracellular pathogens [6]. Th17-related cytokines including IL-17 and IL-23 have been shown to be important for the early control of TB infection [7, 8]. However, it has recently been reported that the level of Th17 cells becomes lower in patients with TB infection [9], and the reason is not clear in regard to programmed cell death.

*M. tuberculosis* infection leads to the apoptosis of CD4^+^ T lymphocytes through interactions between programmed cell death ligand-1 (PD-L1) from dendritic cells and PD-1 on T cells [10]. PD-1 is a member of the extended CD28 family of T cell regulators, and the intracellular tail contains two phosphorylation sites located in an immunoreceptor, which negatively regulate signals from T cell receptors [11]. The percentage of PD-1 on CD4 T lymphocytes has been reported to be higher in patients with active TB, and that this may induce T cell malfunction [12]. In Th17 cells, the roles of PD-1, PD-L1 and other suppressing cells, like Th2 and Treg cells, in active TB have yet to be elucidated. Therefore, we conducted the present study to investigate associations between the percentages of PD-1 and PD-L1 and changes in Th17 cells in patients with active TB.

## Methods

### Patient enrollment

This prospective study was conducted at National Taiwan University Hospital from January 2014 to August 2016. Patients aged ≥20 years who were diagnosed with active TB were recruited. Active TB was diagnosed by cultures positive for *Mycobacterium tuberculosis* or a typical pathology of Mycobacterium tuberculosis infection or suspicious radiographic findings plus a positive response to empirical TB treatment [4, 13]. In addition, we recruited age- and sex-matched controls with negative sputum cultures for mycobacteria. Patients with human immunodeficiency virus (HIV) infection, autoimmune diseases under regular chemotherapy, and those with a bleeding tendency that increased the risk of blood sampling were excluded.

The Research Ethics Committee of National Taiwan University Hospital approved this study (IRB No: 201312043RINB). All of the participants provided written informed consent, and the methods were carried out in accordance with the approved guidelines.

### Isolation of peripheral blood mononuclear cells (PBMCs) or lymphocytes

Peripheral blood from the enrolled subjects was sampled into heparin-containing tubes. Mononuclear cells were immediately isolated using Ficoll-Paque PLUS (GE Healthcare Life Sciences, Sweden), and were then suspended in medium containing RPMI-1640 (Life Technologies; USA), 10% fetal bovine serum (FBS), and 1% penicillin-streptomycin (Life Technologies, USA). We isolated lymphocytes by negative selection using CD14-positive selection system (MACS system, Miltneyi Biotec Inc.) if PBMCs was more than 5 x 10^6^ cell/mm^3^. Otherwise, we used PBMCs for further experiments for avoiding cells loss during lymphocyte selection. The intracellular cytokine responses in CD14-negative lymphocytes were similar to those in PBMCs, which were lower than those in peripheral blood leukocytes (Additional file 1: Figure S1, supplement file). All cells were immediately frozen using a CELL-BANKER system (ZENOAQ, Japan) following the manufacturer’s instructions. The cells were then stored at -80 °C and defrosted within days of the scheduled experiments. Before the experiments, viable cells were counted using a Scepter™ 2.0 Handheld Automated Cell Counter (Millipore Corporation, Billerica, MA, USA).

### RNA isolation and real-time polymerase chain reaction

Total cellular RNA was extracted from peripheral blood lymphocytes after 6 h of stimulation with phorbol 12-myristate 13-acetate (50 uM) and Ionomycin (10 uM) using a Direct-zol™ RNA MiniPrep kit (Zymo Research, CA, USA) according to the manufacturer’s instructions. First-strand cDNA was synthesized using an iScriptTM cDNA Synthesis kit (Life Science Research, USA). A real-time polymerase chain reaction (PCR) with iQ SYBR Green Supermix (BIO-RAD, Singapore) was performed with 4.5 ng of cDNA using a Bio-Rad MyiQ Single-Color Real-Time PCR Detection System, and analyzed using Bio-Rad iQ5 Optical System 2.0 (Bio-Rad, CA). PCR mixtures were denatured at 95 °C for 5 min, followed by 40 cycles of 15 s at 95 °C, 30 s at 58 °C, and 30 s at 72 °C for amplification. The sense and antisense primers of IFN-γ (forward, 5’-ATT CGG TAA CTG ACT TGA ATG TCC-3’; reverse, 5’-CTC TTC GAC CTC GAA ACA GC-3’), IL-17 (forward, 5’-ATC TCC ACC GCA ATG AGG AC-3’; reverse, 5’-GTG G.AC AAT CGG GGT GACAC-3’), and GAPDH (forward, 5’-CCT CAA GAT CAT CAG CAA TG-3’; reverse, 5’-CAC GAT ACC AAA GTT GTC AT -3’) were applied. The mRNA expression level of each target gene was normalized to the respective GAPDH expression.

### Flow cytometry for PBMCs or peripheral blood lymphocytes

The PBMCs or peripheral blood lymphocytes were cultivated in 96-well plates in 300 μl of RPMI 1640 medium with 10% Fetal bovine serum and 1% penicillin-streptomycin (2 x 10^5^ cells per well). Phorbol 12-myristate 13-acetate (50 ng/ml, TOCRIS, USA), and ionomycin calcium salt (1 μM/ml, Sigma-Aldrich, USA) were added to enhance the levels of cytokines because intracellular cytokine staining yielded a very low percentage (Additional file 1: Figure S1, supplement file) at baseline without stimulation. Protein transport inhibitor (BD Bioscience, USA) was added to the co-culture. The peripheral blood lymphocytes were retrieved after 6 h of reaction and measured using flow cytometry (FACSVerse, BD Biosciences, USA). We measured the level of CD4^+^ T cells using anti-CD4-APC, and stained different types of T cells with anti-IFN-γ-PerCP, anti-IL-4-PerCP, anti-17A-PE, anti-CD25-FITC, anti-Foxp3-PerCP, anti-TNF-α-PerCP antibodies (BD Biosciences, CA, USA). Anti-PD-1-PEcy7.0 (used together with anti-IFN and anti-IL-17A), anti-PD-1-PE (used for other staining antibodies), anti-PD-L1-FITC, and anti-cytotoxic T-lymphocyte associated protein 4 (CTLA-4)-PEcy7.0 (eBiosciences, USA) were used to examine the percentages of PD-1, PD-L1 and CTLA-4 on T cells. Data were analyzed using BD FACSuite V software (BD, Biosciences, USA). We discriminated the lymphocyte population using forward scatter (FSC) and side scatter (SSC). Within the group, we gated the subgroups of different T lymphocyte and measured the percentage of PD-1, PD-L1 and CTLA-4 respectively.

### Data collection and statistical analysis

Clinical data including age, sex, co-morbidities, radiographic findings and laboratory data at enrollment were recorded in a standardized case report form with default options. Inter-group differences were analyzed using the Student’s *t*-test or Mann-Whitney *U* test for numerical variables, where appropriate. The chi-square test was used for categorical variables. Statistical significance was set at *p* < 0.05. All analyses were performed using SPSS version 19.0 (Chicago, IL).

## Results

A total of 27 patients (20 [74%] males) with active TB were enrolled, with an average age of 50.1 years (standard deviation [SD]: 16.8). We also enrolled 20 controls (12 [60%] males), with an average age of 56.1 (SD: 12.5) years. There were no significant differences in age and sex between the TB and control groups (*p* = 0.193, and 0.317, respectively). Among the TB group, 18 (67%) patients had pulmonary TB, six (22%) had concurrent pulmonary and extra-pulmonary TB, and three (11%) had extra-pulmonary TB only. With regards to the patients with extra-pulmonary TB, five had lymphadenitis, three had pleurisy, and one had spleen involvement. With regards to the diagnosis of TB, 20 patients had culture-confirmed TB including 10 with a typical pathology for TB, six were diagnosed according to a typical pathology, and the remaining one by typical chest imaging and response to empirical TB treatment. Four patients had an underlying malignancy, and five had diabetes mellitus.

**Fig. 1:**
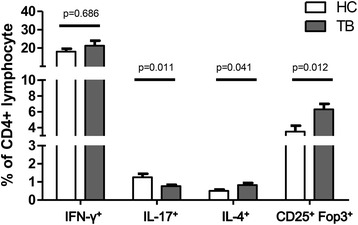
The percentages of T helper (Th) 1 (CD4^+^IFN-γ^+^), Th2 (CD4^+^IL-4γ^+^), Th17 (CD4^+^IL-17A^+^) and regulatory T (Treg) cells (CD4^+^CD25^+^Foxp3^+^) in CD4 positive lymphocytes were measured using flow cytometry. Error bars indicate standard error. Comparisons between the tuberculosis (TB) and control groups were performed using the Mann Whitney *U* test

**Fig. 2:**
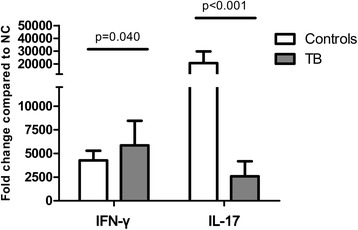
The percentages of interferon-gamma (IFN-γ) and interleukin (IL)-17 were measured using real-time polymerase chain reaction. Fold changes were compared between the cells stimulated by phorbol 12-myristate 13-acetate (50 uM), and Ionomycin (10 uM) and negative controls (mock stimulation). Error bars indicate standard error. Comparisons between groups were performed using the Mann Whitney *U* test

Peripheral blood lymphocytes were used in 21 (78%) and 16 (80%) in TB patients and controls, respectively (*p* = 0.447 by Chi square test). The patients with active TB had an average proportion of 51.8% (SD: 13.5%) CD4-positive lymphocytes, similar to the controls (48.8% [13.1%], *p* = 0.444). Within the CD4-positive lymphocytes, the percentage of Th1 cells, defined as lymphocytes positive for both CD4 and IFN-γ, was comparable between the two groups (21.1% vs. 18.0%, *p* = 0.686) while the percentage of Th2 cells, defined as those positive for both CD4 and IL-4, was higher in the TB group (0.83% vs. 0.51%, *p* = 0.041) (Fig. 1). With regards to Th17 cells, defined as those positive for both CD4 and IL-17A, a lower percentage was noted in the patients with TB than in the controls (1.26% vs. 0.77%, *p* = 0.011). In addition, the percentage of Foxp3 and CD25 in CD4 lymphocytes was higher in the TB group compared with the control group (6.31% vs. 3.50%, *p* = 0.012). With regards to mRNA (as assessed by real-time PCR), the percentage of IFN-γ was slightly higher in the TB group (*n* = 22) than in the controls (*n* = 16) with borderline significance (5859- vs. 4270-fold increase compared to negative control, *p* = 0.040), whereas the percentage of IL-17 was significantly lower in the TB group (2584- vs. 20641-fold increase, *p* < 0.001) (Fig. 2).

**Fig. 3:**
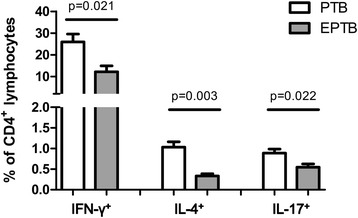
The percentages of T helper (Th) 1 (CD4^+^IFN-γ^+^), Th2 (CD4^+^IL-4γ^+^), and Th17 (CD4^+^IL-17A^+^) in CD4 positive lymphocytes were measured using flow cytometry in patients with pulmonary tuberculosis (PTB) and those with extra-pulmonary TB (EPTB). Error bars indicate standard error. Comparisons between groups were performed using the Mann Whitney *U* test

**Fig. 4:**
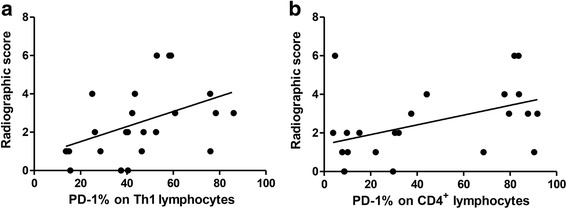
Correlations between lung radiographic score and programmed cell death 1 (PD-1) on (**a**) T helper (Th) 1 lymphocytes or (**b**) CD4^+^ lymphocytes

With regards to the influence of disease extent, the nine patients with extra-pulmonary TB involvement had a lower percentage of Th1 cells (12.2% vs. 25.9%, *p* = 0.021), Th2 cells (0.33% vs. 1.03%, *p* = 0.003), and Th17 cells (0.55% vs. 0.89%, *p* = 0.022) than the 18 patients with pulmonary TB (Fig. 3). The percentage of Treg cells after stimulation was similar between those with or without extra-pulmonary TB involvement (6.88% vs. 6.23%, *p* = 0.679). With regards to the clinical characteristics, more patients with extra-pulmonary TB had cancer (33% vs. 5%, *p* = 0.047), however fewer had diabetes mellitus (0 vs. 26%, *p* = 0.090). When we used the radiographic score to represent the extent or severity of pulmonary TB, we found that it was positively correlated with the percentage of PD-1 on CD4 lymphocytes (Pearson correlation: 0.458, *p* = 0.021) or Th1 cells (CD4^+^ and IFN-γ^+^) (Pearson correlation: 0.446, *p* = 0.025) (Fig. 4), although the percentage of PD-1 on lymphocytes was not significantly different between the TB and control groups.

**Fig. 5:**
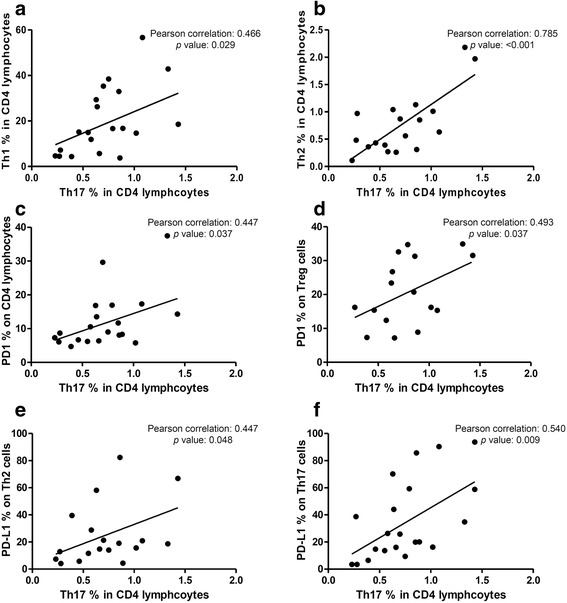
Among the patients with tuberculosis, correlations between the percentage of T helper (Th) 17 cells in CD4 lymphocytes and (**a**) the percentage of Th1 cells in CD4 lymphocytes, (**b**) Th2 cells in CD4 lymphocytes, (**c**) programmed death-1 (PD-1) on CD4 lymphocytes, (**d**) PD-1 on regulatory T cells (Treg), (**e**) PD-1 ligand-1 (PD-L1) on Th2 cells, or (**f**) PD-L1 on Th17 cells

For the variation in Th17 cells in the TB group, the percentage of Th17 cells in the CD4 lymphocytes was positively correlated with the percentage of Th1 (Pearson correlation: 0.466, *p* = 0.029) in the CD4 lymphocytes, Th2 (Pearson correlation: 0.785, *p* < 0.001) cells in CD4 lymphocytes, PD-1 in CD4 lymphocytes (Pearson correlation: 0.447, *p* = 0.037), PD-1 in Treg cells (Pearson correlation: 0.493, *p* = 0.037), PD-L1 in Th2 (Pearson correlation: 0.447, *p* = 0.048), and PD-L1 in Th17 (Pearson correlation: 0.540, *p* = 0.009) cells (Fig. 5).

**Fig. 6:**
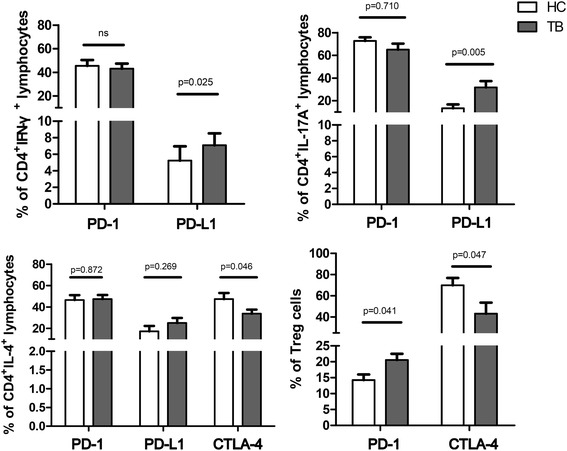
Programmed cell death-1 (PD-1), PD-ligand (PD-L)-1 and cytotoxic T-lymphocyte-associated protein 4 (CTLA-4) percentage in peripheral blood lymphocytes using flow cytometry. Error bars indicate standard error. Comparisons between the tuberculosis (TB) and controls groups were performed using the Mann Whitney *U* test. ns, not significantly different

There was no significant difference in the percentage of PD-1 in Th1 and Th17 cells between the two groups, however the percentage of PD-L1 was higher in Th1 (7.08% vs. 5.23%, *p* = 0.025) and Th17 (31.79% vs. 13.35%, *p* = 0.005) cells in the TB group than in the controls (Fig. 6). No staining for CTLA-4 was performed in Th1 and Th17 cells due to panel and cell limitations. The percentage of CTLA-4 in the Th2 cells was higher in the controls than in the TB group (47.34% vs. 33.74%, *p* = 0.046). In contrast, the percentages of PD-1 and PD-L1 in the Th2 cells were not significantly different between the TB and control groups (Fig. 6). With regards to Treg cells, the percentage of PD-1 was higher (20.56% vs. 14.26%, *p* = 0.041) but that of CLTA-4 (43.23% vs. 69.99%, *p* = 0.047) was lower in the TB group compared to the controls. However, we did not measure the percentage of PD-L1 in Treg cells in either group due to cell limitations (Fig. 6).

## Discussion

In the present study, we observed changes in T lymphocytes in patients with active TB infection, and found a lower percentage Th17 cells but higher percentages of Th2 and Treg cells in the patients with active TB infection compared to the controls. In addition, the patients with extra-pulmonary TB had lower percentages of Th1, Th2, and Th17 cells but a similar percentage of Treg cells compared to those with pulmonary TB. The radiographic score was correlated with the percentage of PD-1 in CD4 or PD-1 on Th1 lymphocytes. With regards to the inhibitory effect of T cell receptors, the percentage of PD-L1 was higher in Th1 and Th17 cells, CTLA-4 was lower in Th2 and Treg cells, and PD-1 was higher in Treg cells in the patients with TB compared to the controls.

Th17 cells are classified as a CD4 T cell subset and are distinct from Th1 and Th2 subsets. Th17 cells produce the cytokines IL-17A and IL-17 F and can lead to a significant pro-inflammatory effect [14], and also protect against intracellular pathogens [15]. Recent studies have reported that Th17 cells are involved in immunity against *M. tuberculosis* [16]. However, the results of Th17 responses in the patients with TB are still inconsistent [17]. A similar IL-17 mRNA expression has been observed in CD4+ blood cells between TB patients and healthy controls [18], and a higher percentage of IL-17^+^CD4^+^ lymphocytes at baseline [19] or after *M. tuberculosis* antigen stimulation has been observed in TB patients [20], whereas a lower percentage of blood IL-17^+^CD4^+^ cells has also been reported in TB patients [9, 16], similar with the present study. These discrepancies might be due to differences in ethnicity, stimulation antigen, or underlying host immunity and require future investigation.

Because a low level of IL-17 in the blood has been associated with a high rate of mortality in TB patients [21] and our observations showed attenuated IL-17^+^CD4^+^ cells in TB patients, we aimed to elucidate the reason for the attenuation of Th17 in TB. A reduced expression of IL-6 receptors on T cells has been postulated [9], and also that the Th17 response may be regulated by signal transducer and activator of transcription 3 and PD-1 signaling [22]. However, associations between Th17 and PD-L1, Th2 and Treg cells have rarely been reported in TB.

In the present study, the percentage of Th17 was lower in the patients with active TB. Interestingly, the percentage of PD-1 in Th17 cells was similar between the TB and control groups, but PD-L1 was higher in the TB group. Although the percentages of PD-1 and PD-L1 have both been reported to be higher in CD4 lymphocytes in patients with TB [12], the percentage of PD-L1 has rarely been discussed in Th17 cells. In chronic mycobacterial infection, the PD-1 pathway, including PD-1 and its ligands, is induced and negatively regulates inflammatory processes [11, 23]. The increase in PD-L1 in Th17 cells may represent self-suppression among lymphocytes in the PD-1 pathway, as previously reported [22].

The percentage of Th2 cells was higher in the TB patients in this study, and was significantly correlated with Th17 cells in the TB group (Pearson correlation: 0.785, *p* < 0.001). The anti-inflammatory effect of Th2 cells may passively increase to counteract the induction of Th1/Th17 during TB infection, and the increase in PD-L1 in Th2 cells may be associated with the suppressive ability of Th2 cells [24]. The trend of an increased percentage of Treg cells from the controls to the patients with TB was in contrast to the Th17 cells. Although the percentage of Treg cells was not correlated with Th17 cells, the percentage of PD-1 in Treg cells was well correlated with Th17 cells. The over-percentage of PD-1 in Treg cells can lead to enhanced function and population expansion in patients with TB [25], and this may play a suppressive role to attenuate the percentage of Th17 cells. In contrast, the percentage of CTLA-4 was lower in the Treg cells in the TB group leading to a less negative signaling, which also induced an expansion in its population [26].

With regards to the extent of disease, the percentages of Th1, Th2 and Th17 cells were lower in the patients with extra-pulmonary TB compared to those with pulmonary TB (Fig. 3) and might influenced the overall results in TB patients. The lower expression of Th1 cells in extra-pulmonary TB is not consistent with previous reports [27, 28] and might be explained by that more patients with extra-pulmonary TB had cancer in this study. On the other hand, the percentage of PD-1 has been reported to be higher in CD4 lymphocytes in patients with TB [12], although this was not observed in the present study. Possible explanations for this include the small number of cases, differences in ethnicity, and a higher number of cases diagnosed by pathology. In addition, 80% of samples in this study were blood lymphocytes and we used stimulation of PMA plus ionomycin, the results might not be compared similarly with previous study. However, the percentage of PD-1 in CD4 or Th1 lymphocytes was still associated with a high extent of disease, as defined by a higher radiographic score. This implies that the percentage of PD-1 is induced by TB bacilli, and that it increases with disease progression.

There are several limitations to the present study. First, the number of cases was small, and making a firm conclusion and performing regression analysis were not possible. Second, patients had comorbidities of cancer and diabetes mellitus were included and the disease spectrum was heterogeneous Both factors might have influenced the immune responses [27–29]. Third, around 20% samples were PBMCs and might influence the results though there was not intergroup difference. However, the cytokine responses were higher in peripheral blood leukocytes than in PBMCs, and these results may have underestimated the cell responses. Last, the role of PD and PD-L1 on T helper cells remains unclear, and future investigations with blocking assays are required.

## Conclusions

The percentage of Th17 cells was decreased in patients with TB, and an increased percentage of PD-L1 on Th17 cells was observed together with higher percentages of Th2 and Treg cells, which might be associated with the attenuation of Th17 cells. Further studies investigating the mechanism of PD-L1 in Th17 cells would be helpful to clarify the pathogenesis of TB. On the other hand, increasing percentage of PD-1 but decreasing CTLA-4 might be responsible for increasing Treg cells whereas lower CTLA-4 might correlate with higher Th2 cells in patients with TB. With regards to the extent of disease, the patients with extra-pulmonary TB had lower percentages of Th1, Th2, and Th17 cells and the patients with more pulmonary involvement had a higher percentage of PD-1 in Th1 lymphocytes. This suggests a complex immune response in patients with TB, and that the percentage of PD-1 in Th1 cells is important within TB process.

## Additional file

Additional file 1: Figure S1. Levels of the intracellular cytokines interferon-gamma (IFN-γ) and interleukin-17 (IL-17) were measured in different cell samples from the same subjects after they were assayed with phorbol 12-myristate 13-acetate (PMA) and ionomycin for 6 h. The response in peripheral blood leukocytes (PBLs) was higher than that in peripheral blood mononuclear cells (PBMCs), which was similar to that in CD14-negative cells. Sti, stimulation. The stimulation of PMA in the figures refers to PMA plus ionomycin. (DOCX 182 kb)
