# Supplementary material for: Decreased T helper 17 cells in tuberculosis is associated with increased percentages of programmed death ligand 1, T helper 2 and regulatory T cells
Source: Respir Res. 2017 Jun 26;18:128. doi: 10.1186/s12931-017-0580-3 (PMC5485543; doi:10.1186/s12931-017-0580-3)
Supplement: Additional file 1: Figure S1. — Levels of the intracellular cytokines interferon-gamma (IFN-γ) and interleukin-17 (IL-17) were measured in different cell samples from the same subjects after they were assayed with phorbol 12-myristate 13-acetate (PMA) and ionomycin for 6 h. The response in peripheral blood leukocytes (PBLs) was higher than that in peripheral blood mononuclear cells (PBMCs), which was similar to that in CD14-negative cells. Sti, stimulation. The stimulation of PMA in the figures refers to PMA plus ionomycin. (DOCX 182 kb) [file 12931_2017_580_MOESM1_ESM.docx]

**Figure S1.**

Levels of the intracellular cytokines interferon-gamma (IFN-γ) and interleukin-17 (IL-17) were measured in different cell samples from the same subjects after they were assayed with phorbol 12-myristate 13-acetate (PMA) and ionomycin for 6 hours. The response in peripheral blood leukocytes (PBLs) was higher than that in peripheral blood mononuclear cells (PBMCs), which was similar to that in CD14-negative cells. Sti, stimulation. The stimulation of PMA in the figures refers to PMA plus ionomycin.
